# Supplementary material for: Mechanism of TCF21 Downregulation Leading to Immunosuppression of Tumor-Associated Macrophages in Non-Small Cell Lung Cancer
Source: Pharmaceutics. 2023 Sep 7;15(9):2295. doi: 10.3390/pharmaceutics15092295 (PMC10536982; doi:10.3390/pharmaceutics15092295)
Supplement: Supplementary file 1 [file pharmaceutics-15-02295-s001.zip › Legend of supplementary figure.pdf]

Figure S1. Macrophage polarization-associated markers detected by flow cytometry. (A) Percentage of CD86 positive expression. (B) Percentage of CD163 positive expression. (C) Percentage of CD206 positive expression. (D) Percentage of HLADR positive expression. Note: *P < 0.05 represents a statistical difference, **P < 0.01 represents a significant statistical difference.

Figure S2. TCF21 acted with Notch4 on the polarization of macrophages. (A) Percentage of CD86 positive expression. (B) Percentage of CD163 positive expression. (C) Percentage of CD206 positive expression. (D) Percentage of HLADR positive expression. Note: *P < 0.05 represents a statistical difference, **P < 0.01 represents a significant statistical difference.
