# Supplementary figures and images for: Mechanism of TCF21 Downregulation Leading to Immunosuppression of Tumor-Associated Macrophages in Non-Small Cell Lung Cancer
Source: Pharmaceutics. 2023 Sep 7;15(9):2295. doi: 10.3390/pharmaceutics15092295 (PMC10536982; doi:10.3390/pharmaceutics15092295)

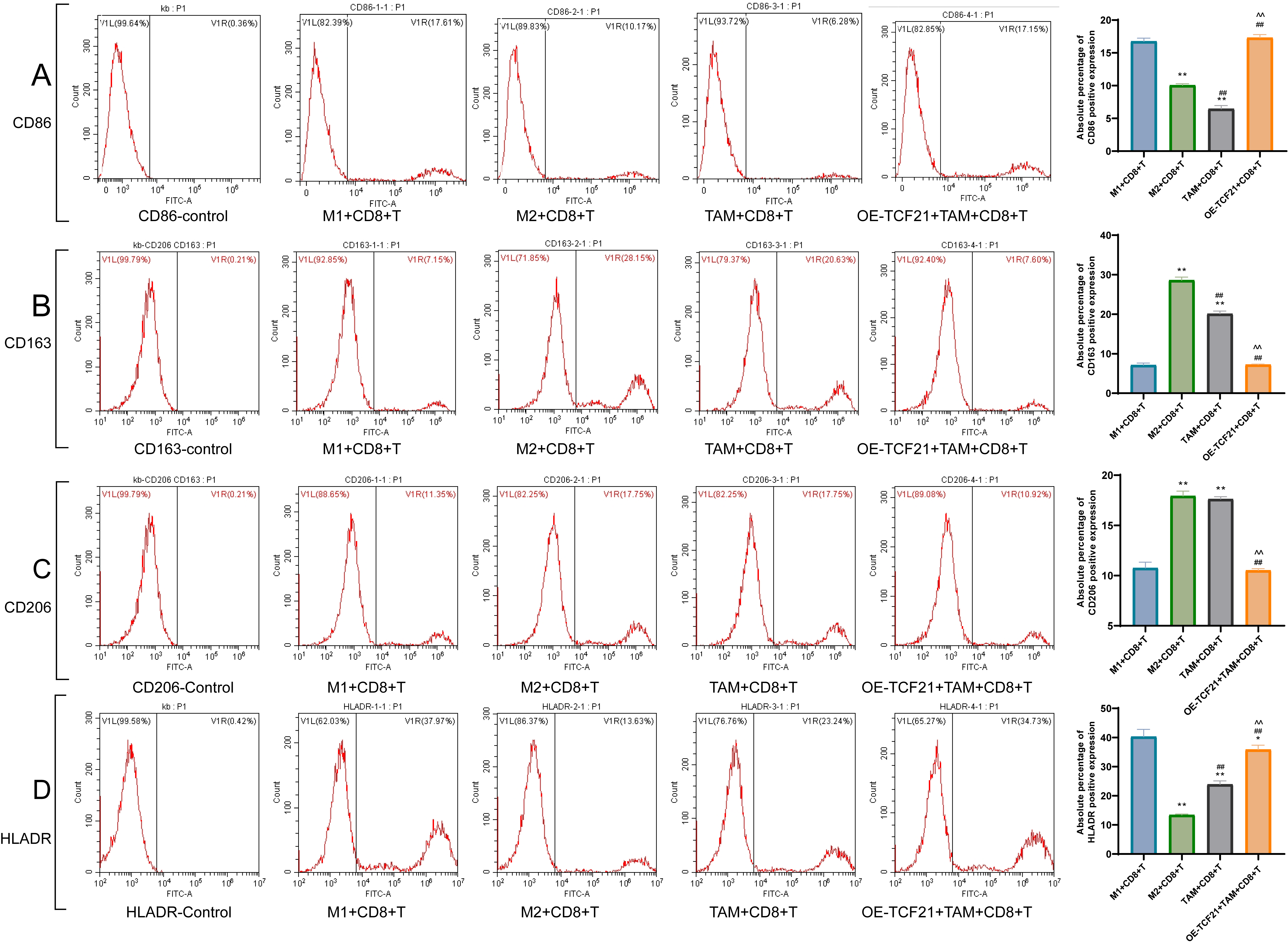

Supplement: Supplementary file 1 [file pharmaceutics-15-02295-s001.zip › S1.tif]

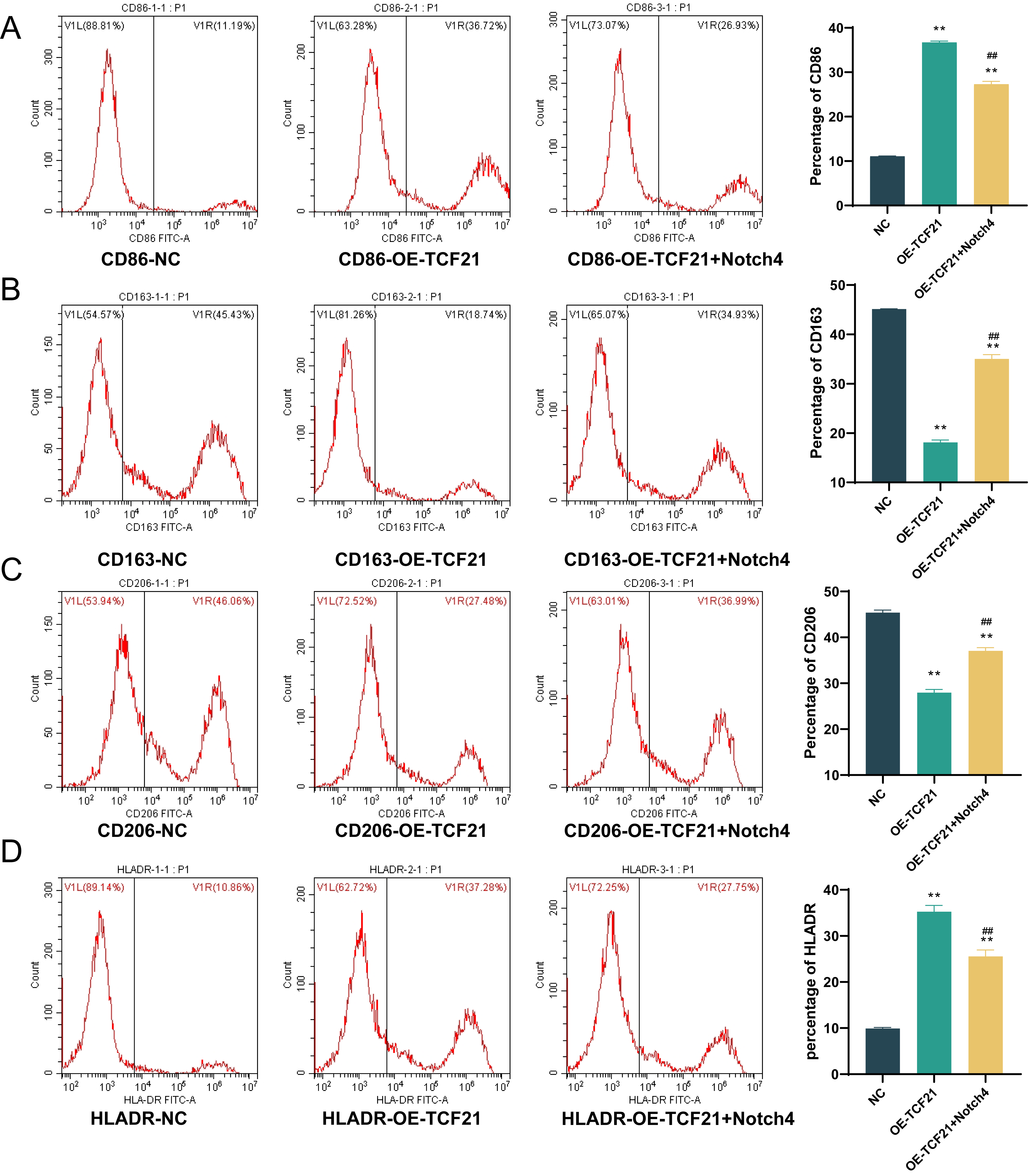

Supplement: Supplementary file 1 [file pharmaceutics-15-02295-s001.zip › S2.tif]
